# Efficacy of twice-daily high-frequency repetitive transcranial magnetic stimulation on associative memory

**DOI:** 10.3389/fnhum.2022.973298

**Published:** 2022-10-12

**Authors:** Qiang Hua, Yuanyuan Zhang, Qianqian Li, Xiaoran Gao, Rongrong Du, Yingru Wang, Qian Zhou, Ting Zhang, Jinmei Sun, Lei Zhang, Gong-jun Ji, Kai Wang

**Affiliations:** ^1^Department of Neurology, The First Affiliated Hospital of Anhui Medical University, Anhui Medical University, Hefei, China; ^2^School of Mental Health and Psychological Sciences, Anhui Medical University, Hefei, China; ^3^Anhui Province Key Laboratory of Cognition and Neuropsychiatric Disorders, Hefei, China; ^4^Collaborative Innovation Centre of Neuropsychiatric Disorder and Mental Health, Hefei, China; ^5^Department of Psychiatry, The First Affiliated Hospital of Anhui Medical University, Hefei, China; ^6^Hefei Comprehensive National Science Center, Institute of Artificial Intelligence, Hefei, China; ^7^Anhui Institute of Translational Medicine, Hefei, China

**Keywords:** associative memory, hippocampal-cortical network, inferior parietal lobule, stimulation dose, transcranial magnetic stimulation

## Abstract

**Objectives:**

Several studies have examined the effects of repetitive transcranial magnetic stimulation (rTMS) on associative memory (AM) but findings were inconsistent. Here, we aimed to test whether twice-daily rTMS could significantly improve AM.

**Methods:**

In this single-blind, sham-controlled experiment, 40 participants were randomized to receive twice-daily sham or real rTMS sessions for five consecutive days (a total of 16,000 pulses). The stimulation target in left inferior parietal lobule (IPL) exhibiting peak functional connectivity to the left hippocampus was individually defined for each participant. Participants completed both a picture-cued word association task and Stroop test at baseline and 1 day after the final real or sham rTMS session. Effects of twice-daily rTMS on AM and Stroop test performance were compared using two-way repeated measures analysis of variance with main factors Group (real vs. sham) and Time (baseline vs. post-rTMS).

**Results:**

There was a significant Group × Time interaction effect. AM score was significantly enhanced in the twice-daily real group after rTMS, but this difference could not survive the *post hoc* analysis after multiple comparison correction. Further, AM improvement in the twice-daily real group was not superior to a previously reported once-daily rTMS group receiving 8,000 pulses. Then, we combined the twice- and once-daily real groups, and found a significant Group × Time interaction effect. *Post hoc* analysis indicated that the AM score was significantly enhanced in the real group after multiple comparisons correction.

**Conclusion:**

Our prospective experiment did not show significant rTMS effect on AM, but this effect may become significant if more participants could be recruited as revealed by our retrospective analysis.

## Introduction

Transcranial magnetic stimulation (TMS) is a non-invasive technique for modulating brain network connectivity with demonstrated therapeutic efficacy against neurological and neuropsychiatric illnesses (Fox et al., 2012). Wang et al. (2014) reported that once-daily repetitive TMS (rTMS) to the inferior parietal lobule (IPL), a region strongly connected to the hippocampus, significantly improved associative memory (AM) in healthy participants, suggesting possible utility for treatment of disorders characterized by AM deficits, such as stroke, age-related cognitive decline, neurotrauma, and various neuropsychiatric and neurodegenerative conditions.

However, the physiological response to non-invasive brain stimulation is known to be highly variable among individuals (López-Alonso et al., 2014), and several subsequent TMS studies found no significant AM improvement. For instance, a survey of research groups found that approximately 50% were not able to reproduce rTMS effects from original publications and a recent investigation by Héroux et al. (2015) reported significant changes in functional connectivity (FC) following multi-day rTMS of the parietal cortex but no AM enhancement (Hendrikse et al., 2020). Similarly, we found no significant difference in AM following once-daily rTMS sessions for 5 days compared to a sham group (Gao et al., 2021). Collectively, these inconsistencies suggest that rTMS efficacy for improving AM is highly dependent on stimulus protocol (e.g., stimulus intensity, duration), target, study design, and (or) treatment group characteristics.

Increasing the stimulation dose (total number of impulses) is one potential method to achieve a more robust effect on AM (Nettekoven et al., 2014). A recent study found that a high-dose rTMS protocol is safe and produces more reliable remission from depression (Cole et al., 2020). In addition to stimulation dose, the experimental design may influence outcome. The seminal study by Wang et al. (2014) and most subsequent studies reproducing AM improvement (Freedberg et al., 2019; Hermiller et al., 2019; Hendrikse et al., 2020; Gao et al., 2021) used a within-subject crossover design with an approximately 2-week delay (washout period) between real and control (sham) stimulation. However, the AM improvement from baseline was still significant 2-weeks after real rTMS, suggesting that a longer interval is needed for crossover studies (Wang and Voss, 2015; Gao et al., 2021). Nonetheless, no study has specifically examined the optimal interval for comparison of control (sham) stimulation to real stimulation.

In the present study, we aimed to investigated whether a higher rTMS dose could produce significantly greater AM improvement, beyond the sham rTMS. To this end, we modified the paradigm of our previous study (Gao et al., 2021) in two points: (1) using a parallel rather than crossover design; (2) doubling the 20-Hz rTMS dose (twice-daily sessions for 5 days, total 16,000 pulses).

## Materials and methods

### Participants

Forty healthy subjects (24 females and 16 males) with no history of rTMS, transcranial electric stimulation, neuropsychological disorders, or psychoactive drug use were recruited for this study. All participants met the safety criteria for MRI and rTMS (Rossi et al., 2009) and provided written informed consent. Each was remunerated for their participation after study completion. Experiments were conducted in accordance with the Declaration of Helsinki (2008 revised edition) and were approved by the local ethics committee.

### Experimental design

This was a randomized, single-blind, sham-controlled, parallel design study consisting of two arms, real 20-Hz rTMS over the IPL (experimental) and sham rTMS (control). Forty subjects were included based on our previous work using single daily rTMS sessions. Subjects were assigned to the real or sham group according to random number selection while ensuring 20 per group. All subjects were unaware of the stimulation protocols until the end of the study (single-blind).

**FIGURE 1:**
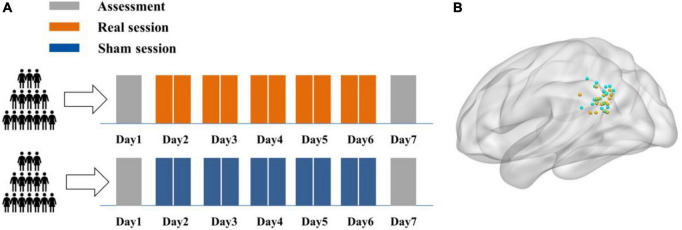
Experimental design. **(A)** Each participant received ten real or sham rTMS sessions delivered two per day for five consecutive days. A face-cued recall (associative memory) task and non-associative Stroop task were completed 1 day prior to the first stimulation session (baseline) and 1 day after the final session. **(B)** The individual stimulation sites in the left IPL were identified by rs-fMRI maps of hippocampal functional connectivity. The stimulation sites for all subjects in real and sham groups are demarcated by green and yellow spheres, respectively, in standard MNI space.

Each participant received twice-daily rTMS sessions over five consecutive days, for a total of ten sessions and 16,000 pulses. A face-cued word recall task was using to test AM and the Stroop test to assess non-associative memory cognitive processing. Tasks were performed both 1 day prior to the first real or sham stimulation session (baseline assessment) and 1 day after the final session (post-rTMS assessment). Structural MRI and rs-fMRI were conducted on each subject prior to baseline testing to identify the IPL target site (Figure 1A). After each session, subjects self-reported TMS adverse events on a numeric rating scale from 0 (no side effects) to 5 (unbearable side effects).

### Magnetic resonance images acquisition

Magnetic resonance images (MRI) were collected at the University of Science and Technology of China (Hefei, Anhui Province) using a 3.0 T scanner (Discovery 750; GE Healthcare, Milwaukee, WI, United States). Functional and structural images were acquired using the same parameters as in our previous studies (Ji et al., 2019a,b, 2021). Briefly, high spatial resolution T1-weighted anatomic images were acquired in the sagittal orientation using a three-dimensional brain-volume sequence (repetition/echo time, 8.16/3.18 ms; flip angle, 12°; field of view, 256 × 256 mm^2^; 256 × 256 matrix; section thickness, 1 mm, without intersection gap; voxel size, 1 × 1 × 1 mm^3^; 188 sections). Following structural MRI scanning, functional images (217 volumes) were acquired using a single-shot gradient-recalled echo planar imaging sequence (repetition/echo time, 2,400/30 ms; flip angle, 90°). Images of 46 transverse sections (field of view, 192 mm × 192 mm; 64 × 64 in-plane matrix; section thickness without intersection gap, 3 mm) were acquired parallel to the anteroposterior commissure line. Foam fillers and earplugs were used to minimize head motion and scanner noise during image acquisition. All participants were asked to keep their eyes close and rest without falling asleep during scanning. Scanning was performed prior to AM and Stroop testing to exclude potential carry-over effects.

### Identification of stimulation locations

Cortical stimulation locations over the IPL were identified from individual resting-state functional connectivity maps with the left hippocampus as the seed region (Wang et al., 2014; Hendrikse et al., 2020; Gao et al., 2021). The hippocampal seed was set at [−24, −18, −18] in Montreal Neurological Institute (MNI) coordinates according to Wang et al. (2014). The stimulation target for each participant was defined as the strongest connectivity site within the spherical mask of the left IPL (MNI coordinates [−47, −68, 36], radius 15 mm).

Preprocessing consisted of seven steps: (1) deleting the first five functional volumes; (2) slice timing correction and realignment; (3) co-registration of structural and functional images; (4) normalization of functional images by the matrix computed in structural segmentation and normalization; (5) smoothing of functional images using a 4-mm full-width at half-maximum isotropic Gaussian kernel; (6) temporal band-pass filtering (0.01–0.1 Hz); (7) regressing out 27 nuisance signals (average white matter, cerebrospinal fluid, and whole-brain signals as well as 24 head motion parameters). All processing steps were performed using Statistical Parametric Mapping (SPM) 12 and in-house software TMStarget.^1,2^

### Transcranial magnetic stimulation parameters

Transcranial magnetic stimulation was delivered to the IPC using a Magstim Rapid^2^ stimulator (Magstim Company, Whitland, United Kingdom) through a 70-mm air-cooled figure-of-eight coil under the guidance of a frameless stereotactic optical tracking neuronavigation system (Brainsight; Rogue Research, Montreal, QC, Canada) (Gao et al., 2021). Individual stimulus intensity was set according to the resting motor threshold (RMT), defined as the minimum stimulator output necessary to evoke a potential with peak-to-peak amplitude ≥50 mV from the right first dorsal interosseous (FDI) muscle in at least 5 out of 10 consecutive trials (Ji et al., 2017). For the real stimulation group, rTMS was applied with 100% of RMT at 20-Hz (2 s on, 28 s off) for 20 min (1,600 pulses/session) over the individual IPL target. A total of 10 rTMS sessions were performed over a 5-day period with 2 sessions per day (16,000 pulses in total) separated by at least 1 h.

For the sham stimulation condition, participants received the same rTMS protocol using a sham coil (Magstim Company, Whitland, United Kingdom) with the same appearance as the real coil to avoid participants identifying rTMS group allocation. This sham coil generated only sound and sensations on the scalp similar to the real coil but no current (Chen et al., 2019).

### Associative memory test

We employed the computerized Chinese face-cued word recall task described by Gao et al. (2021) to assess AM. Each subject studied 15 photographs of Chinese faces presented individually on a computer screen while a common word was read aloud in standard Mandarin. Each face corresponded to a unique word and was shown for 4 s. There were four alternative versions of the test, each using a different set of faces and words, and each participant was randomly assigned two, one to complete at baseline and the other following sham or real rTMS. All the face photos were presented at 4,800 × 6,000 pixels per inch in greyscale and all words were nouns of two Chinese characters taken from the Chinese Corpus Word list, with written frequency between 500 and 3,000 (Chinese Language and Writing Network).^3^ Subjects were instructed to pay attention and try to remember the face-word associations. After the learning phase, subjects were given a rest of approximately 1 min, followed by face ire-presentation in a different and random order. Subjects were instructed to recall the word that accompanied each face during the learning phase, and each word response was scored as correct or incorrect (with no errors relating to pronunciation). Participants received no prompts or feedback on the correctness of their answers. The number of correctly recalled face–word pairs was recorded as the AM score. To account for inter-subject differences in baseline AM performance, the individual improvement in AM following rTMS was expressed as a percentage change relative to baseline [AM score percentage change = *(post correct – baseline correct)/baseline correct* × *100%*] (Wang et al., 2014).

### Stroop test

To prevent the participants from easily guessing the purpose of the study and to assess general non-associative cognitive processing capacity, a Stroop Color Word Test (Victoria version) adapted to local Chinese was also conducted (Lee and Chan, 2000; Lee et al., 2002; Yu and Lee, 2018). The test stimuli included images of colored dots (Part A), words unrelated to color presented in colored font (Part B), and color names presented in font colors different from the word (Part C) (e.g., the word “red” in green font). Each image consisted of 24 items in red, green, blue, or yellow presented in a 4-by-6 matrix. Each color was used six times per image, and the four colors were arranged once per row in a pseudo-random order. The participants were asked to name the colors of stimuli (font) from left to right and from top to bottom while ignoring semantic content (i.e., the correct answer for the example above is “green”). For each condition, the naming completion time (response time) and number of errors were recorded. The interference value was defined as the response time for Part B minus Part A (low interference condition), and Part C minus Part A (high interference condition).

### Methodological similarities and differences from Gao et al. (2021)

Gao et al. (2021) used a within-subjects design to examine the effect of rTMS on AM. Participants received real rTMS on IPL and sham rTMS on pre-SMA targets, separated by at least 2 weeks. For each condition, a total of 5 rTMS sessions were performed over 5 consecutive days with one session per day. The rTMS involving 1,600 total pulses in one session was delivered at 100% of RMT at 20 Hz (2 s followed by 28 s of vacancy). The experimental design of the current study was modified according to Gao et al. (2021). The same rTMS parameters and face-cued word recall task were used in both studies. In contrast to Gao et al. (2021), the current study added a second session to double the rTMS dose (for a total of 16,000 pulses rather than 8,000). Additionally, we used a between-subjects design and sham stimulation was performed at 100% RMT over IPL with a sham coil in the current study.

### Statistical analysis

Continuous baseline variables were compared between groups by independent samples *t*-test and categorical baseline variables by *χ^2^* test. Differences in AM and Stroop test performance were compared by two-way repeated measures analysis of variance (RT-ANOVA) with main factors Group (sham vs. real rTMS) and Time (baseline vs. post-rTMS), followed by *post hoc* Sidak’s multiple comparison tests. AM score percentage change was compared between groups using the independent samples *t*-test. Outliers were identified by non-linear regression using GraphPad Prism and removed from subsequent analysis.

Three separate analyses were performed using two-way ANOVA. The primary analysis included only the current data from participants receiving sham rTMS and participants receiving real rTMS (termed the *Twice-daily dataset* including *Twice-daily(R)* and *Twice-daily(S)* groups). The second analysis tested if the higher rTMS dose produced more prominent effects on AM by comparing the *Twice-daily(R)* group to the *Once-daily(R)* group. This *Once-daily(R)* group included 16 subjects from Gao et al. (2021). In the third analysis, we combined *Twice-daily(R) and Once-daily(R)* groups to produce a *Combined(R)* group and investigated the effects on AM compared to the *Twice-daily(S)* group.

## Results

### Primary analysis of the current experimental cohort

#### Characteristics of participants

Forty subjects were initially recruited to receive twice-daily sham or real rTMS (*Twice-daily dataset*) but one participant randomized to the sham group [*Twice-daily(S)*] dropped out for personal reasons. Thus, data from 39 subjects (23 females/16 males, mean age = 21.69, SEM = 0.37, range 18–29 years) were included in the primary analyzes. *Twice-daily(R) and Twice-daily(S)* groups did not differ significantly in age, gender ratio, RMT, test delay (time between the final stimulation session and post-rTMS test), baseline AM score, or baseline Stroop test score (Table 1). The averaged side effect scores are presented in Table 1, and no significant difference (*p* = 0.12) between the *Twice-daily(R)* and *Twice-daily(S)* groups. In general, both sham and real rTMS were well tolerated, with only slight discomfort reported by some participants. But this effect disappeared after the stimulation ended. The average (± SEM) MNI coordinate of IPL stimulation was *x* = −45.4 (0.92), *y* = −72.0 (0.83), *z* = 33.1 (1.01) (Figure 1B).

**TABLE 1:** Characteristics of participants from twice-daily data.

	*Twice-daily(R)* (*N* = 20)	*Twice-daily(S)* (*N* = 19)	Statistics/p
**Demographic**			
Age (years)	22.25 (0.55)	21.11 (0.48)	0.14^a^
Gender (female/male)	13/7	10/9	0.52^b^
RMT (%)	60.80 (1.23)	63.53 (1.27)	1.54/0.20^c^
Test delay (h)^d^	21.22 (0.82)	21.49 (0.93)	0.21/0.83^c^
**Tests**			
AM test (Baseline)	4.55 (0.45)	5.16 (0.62)	0.80/0.43^c^
Stroop test (Baseline)			
low interference (s)	1.50 (0.28)	0.75 (0.28)	1.88/0.07^c^
high interference (s)	7.62 (0.90)	7.50 (0.66)	0.11/0.91^c^

Data from Twice-daily(R) and Twice-daily(S) groups are represented as mean (SEM). ^a^Mann–Whitney test; ^b^Fisher’s exact test; ^c^Two-sample t-test; ^d^Test delay depicts the interval between the end of final stimulation session and the post-rTMS tests.

#### Associative memory performance

**FIGURE 2:**
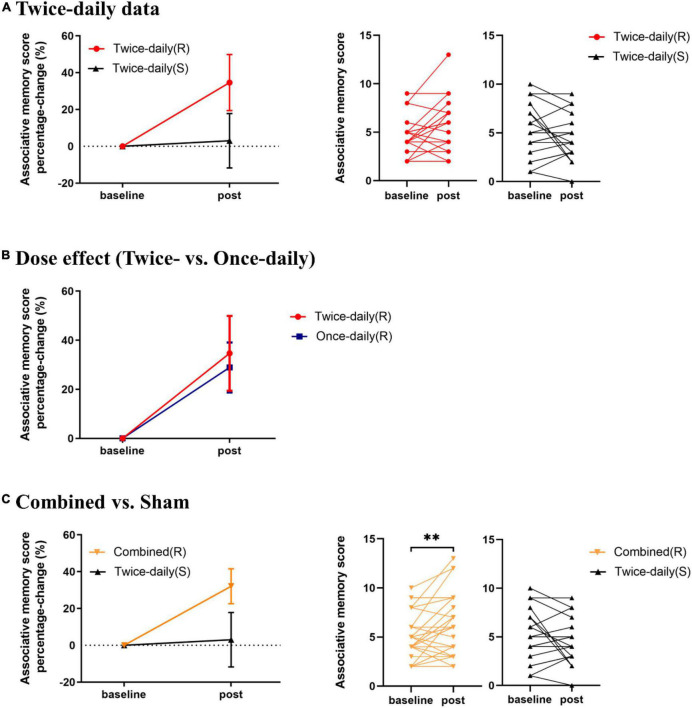
Effects of rTMS on associative memory performance. **(A)** Mean percentage change in AM score from baseline for *Twice-daily(R)* and *Twice-daily(S)* groups. The interaction effect was significant, but *post hoc* analyses indicated no significant increase following real or sham stimulation. **(B)** Mean percentage change in AM score from baseline for *Twice-daily(R)* and *Once-daily(R)* groups. There was no significant Group × Time interaction. **(C)** Mean percentage change in AM score for *Combined(R)* and *Twice-daily(S)* groups. A significant Group × Time interaction was found, and *post hoc* analyses indicated that real rTMS but not sham rTMS significantly improved AM score compared to baseline. AM score Percentage-change = *(post correct – baseline correct)/baseline correct* × *100%*. Error bars indicate SEM, ^**^P < 0.01.

The AM scores for the *Twice-daily(R)* and *Twice-daily(S)* groups are presented in Table 2. Two-way RT-ANOVA revealed a significant Group [*Twice-daily(R)* vs. *Twice-daily(S)*] × Time (baseline vs. post-rTMS) interaction (*F*_1_,_37_ = 5.99, *p* = 0.019), but no main effect of Time (*F*_1_,_37_ = 0.51, *p* = 0.48) or Group (*F*_1_,_37_ = 0.16, *p* = 0.70). *Post hoc* analyses using Sidak’s multiple comparison test indicated that AM score was not increased significantly after real stimulation (*t* = 2.26, *p* = 0.06; Figure 2A) or sham stimulation (*t* = 1.21, *p* = 0.41; Figure 2A) compared to baseline. Further, AM score change normalized to baseline (percentage change) did not differ between *Twice-daily(R)* and *Twice-daily(S)* groups [34.7% (SEM = 15.2) vs. 3.0% (SEM = 14.8); *P* = 0.16].

**TABLE 2:** Associative memory performance.

	AM scores	

	Baseline	Post-rTMS
Twice-daily(R) group	4.55 (0.45)	5.70 (0.63)
Twice-daily(S) group	5.16 (0.62)	4.53 (0.53)
Once-daily(R) group	4.69 (0.62)	5.94 (0.84)
Combined(R) group	4.61 (0.37)	5.81 (0.51)

AM performance provided as mean raw scores (numbers of words correctly recalled). Data are represented as mean (SEM).

#### Stroop test performance

Two measures of Stroop test performance, response time and error rate, are presented in Supplementary Table 1. ANOVA indicated no significant main effects of Group [*Twice-daily(R)* vs. *Twice-daily(S)*] and Time, and no significant Group [*Twice-daily(R)* vs. *Twice-daily(S)*] × Time (baseline vs. post-rTMS) interaction (see details in the Supplementary material).

### Twice-daily versus once-daily repetitive transcranial magnetic stimulation

#### Characteristics of participants

There were no significant differences in age, gender ratio, RMT, AM scores, and Stroop tests between *Twice-daily(R)* and *Once-daily(R)* groups, while test delay was slightly but significantly lower in the *Twice-daily(R)* group (Table 3).

**TABLE 3:** Characteristics of participants from *Twice-daily(R)* and *Once-daily(R)*.

	*Twice-daily(R)* (*N* = 20)	*Once-daily(R)* (*N* = 16)	Statistics/p
**Demographic**			
Age (years)	22.25 (0.55)	21.13 (0.48)	0.25^a^
Gender (female/male)	13/7	9/7	0.31^b^
RMT (%)	60.80 (1.23)	59.50 (2.15)	0.55/059^c^
Test delay (h)^d^	21.22 (0.82)	23.28 (0.17)	2.21/0.03^c^
**Tests**			
AM test (Baseline)	4.55 (0.45)	4.69 (0.62)	0.18/0.86^c^
Stroop test (Baseline)			
low interference (s)	1.50 (0.28)	1.06 (0.59)	0.08^b^
high interference (s)	7.62 (0.90)	7.38 (1.24)	0.16/0.88^c^

Data from Twice-daily(R) and Once-daily(R) are represented as mean (SEM). ^a^Mann–Whitney test; ^b^Fisher’s exact test; ^c^Two-sample t-test; ^d^Test delay depicts the interval between the end of final stimulation session and the post-rTMS tests.

#### Associative memory performance

Analysis of variance revealed no significant Group [*Twice-daily(R)* and *Once-daily(R)*] × Time (baseline and post-rTMS) interaction (*F*_1_,_34_ = 0.02, *p* = 0.89) (Table 2) suggesting no difference in effect stability between *Twice-daily(R)* and *Once-daily(R)* rTMS. There was a significant main effect of Time (*F*_1_,_34_ = 11.96, *p* = 0.002) but not Group (*F*_1_,_34_ = 0.05, *p* = 0.82). Average percentage change in AM score did not differ significantly between *Twice-daily(R)* and *Once-daily(R)* groups [34.7% (SEM = 15.2) vs. 28.9% (SEM = 10.2); *p* = 0.80] (Figure 2B).

#### Stroop test performance

The response times and error rates for the Stroop tests from *Once-daily(R)* and *Twice-daily(R)* groups are presented in Supplementary Table 1. ANOVA revealed no significant interaction of Group [*Twice-daily(R)* vs. *Once-daily(R)*] × Time (baseline vs. post-rTMS). See details in the Supplementary material.

### Combined analysis of once-daily and twice-daily repetitive transcranial magnetic stimulation

#### Characteristics of participants

There were no significant differences in any of the aforementioned baseline characteristics between *Combined(R)* and *Twice-daily(S)* groups (Table 4).

**TABLE 4:** Characteristics of participants from *Combined(R)* and *Twice-daily(S)*.

	*Combined(R)* (*N* = 36)	*Twice-daily(S)* (*N* = 19)	Statistics/*p*
**Demographic**			
Age (years)	21.75 (0.38)	21.11 (0.48)	0.35^a^
Gender (female/male)	20/16	10/9	>0.9999^b^
RMT (%)	60.22 (1.16)	63.53 (1.27)	1.79/0.08^c^
Test delay (h)^d^	22.14 (0.49)	21.42 (0.92)	0.68/0.50^c^
**Tests**			
AM test (Baseline)	4.61 (0.37)	5.16 (0.62)	0.81/0.42^c^
Stroop test (Baseline)			
low interference (s)	1.31 (0.30)	0.75 (0.28)	1.23/0.22^c^
high interference (s)	7.51 (0.74)	7.50 (0.66)	0.01/0.99^c^

Data from Combined(R) and Twice-daily(S) groups are represented as mean (SEM). ^a^Mann–Whitney test; ^b^Fisher’s exact test; ^c^Two-sample t-test; ^d^Test delay depicts the interval between the end of final stimulation session and the post-rTMS tests.

#### Associative memory performance

Associative memory scores for the *Combined(R)* and *Twice-daily(S)* groups are presented in Table 2. ANOVA revealed a significant Group [*Combined(R)* vs. *Twice-daily(S)*] × Time (baseline vs. post-rTMS) interaction effect on AM change (*F*_1_,_53_ = 9.08, *p* = 0.004) but no main effect of Time (*F*_1_,_53_ = 0.86, *p* = 0.36) or Group (*F*_1_,_53_ = 0.29, *p* = 0.59). Further, *post hoc* analyses using Sidak’s multiple comparison tests indicated that real stimulation significantly enhanced AM score compared to baseline (*t* = 3.35, *p* = 0.003) (Figure 2C) while sham stimulation did not (*t* = 1.29, *p* = 0.37) (Figure 2C). In addition, the average percentage change in AM score was not significantly higher in the *Combined(R)* group than the *Twice-daily(S)* group [32.11% (SEM = 9.49) vs. 3.0% (SEM = 14.8); *p* = 0.09] (Figure 2C).

#### Stroop test performance

The Stroop test response times and error rates for *Combined(R)* and *Twice-daily(S)* groups are presented in Supplementary Table 1. ANOVA indicated no significant Group [*Combined(R)* vs. *Twice-daily(S)*] × Time (baseline vs. post-rTMS) interaction. See details in the Supplementary material.

## Discussion

In the present study, we found that the twice-daily rTMS enhanced AM significantly better than the placebo effect. However, the improvement in the twice-daily real stimulation group was not significant after multiple comparisons correction. Furthermore, the twice-daily protocol was not superior to the once-daily sessions previously reported by Gao et al. (2021). When combining the twice- and once-daily rTMS data to enlarge sample size, the group by time interaction effect showed better AM improvement in the real than sham group, and the *post hoc* analysis in real group survived the multiple comparisons correction.

Wang et al. (2014) first reported that once-daily rTMS session targeting the hippocampal-cortical network can significantly improve AM, but several subsequent studies yielded inconsistent findings. We speculated that doubling the number of stimuli by delivery twice-daily rTMS sessions could induce significant AM improvement as has been shown for other clinical effects of rTMS, including relief of depressive symptoms (Cole et al., 2020). Twice-daily rTMS sessions did enhance AM task performance to a greater extent than sham rTMS (34.7 vs. 3.0%). But the difference from baseline was not significant by *post hoc* multiple comparison tests for the twice-daily real stimulation group. As small sample size reduces statistical power (Button et al., 2013), we combined the *Twice-daily dataset* and *Once-daily dataset* from our previous experiment (Gao et al., 2021) to enlarge the sample size (*N* = 36), and *post hoc* multiple comparison test analyses showed significant AM improvement. An insufficient sample size could contribute to variability and affect the reliability of non-invasive brain stimulation studies (Guerra et al., 2020a,b). Thus, the required sample size should be calculated with great care in future TMS studies. We recommend that future studies examining multi-day rTMS-induced effects on memory include more participants in the real rTMS group.

Increasing the number of rTMS sessions per day and number of pulses per day has been reported to have superior antidepressant and altering cortical excitability efficacy (Nettekoven et al., 2014; Cole et al., 2020). Thus, increasing the session number was expected to enhance AM improvement from rTMS on the IPL. However, improvements twice-daily and once-daily rTMS were roughly equal (34.7 vs. 28.9%). An alternative strategy to increase rTMS dose is to use multiple targets, as several studies have demonstrated that other cortical nodes in the hippocampal-cortical network, such as dorsolateral prefrontal cortex (DLPFC), contribute to AM (Blumenfeld and Ranganath, 2006; Bilek et al., 2013). Future studies should consider combined DLPFC- and IPL-targeted rTMS.

This study has several limitations, most notably the small sample size. Although combining datasets yielded significant AM improvement, the two studies differed in several respects (e.g., experimental design, stimulus doses). Future large-sample prospective studies are warranted to document the reliability and duration of this AM-enhancing effect. Second, the RMT was measured only once rather than prior to each session, and a previous study reported that RMT varied significantly across days among subjects receiving rTMS (Cotovio et al., 2021).

## Conclusion

Possibly due to the small sample size, our prospective experiment did not find significant rTMS effect on memory. But this effect may become significant if more participants could be recruited as revealed by our retrospective analysis.

## Data availability statement

The raw data supporting the conclusions of this article will be made available by the authors, without undue reservation.

## Ethics statement

The studies involving human participants were reviewed and approved by the Anhui Medical University. The patients/participants provided their written informed consent to participate in this study.

## Author contributions

QH, G-JJ, and KW: conceptualization. QH, YZ, QL, XG, RD, YW, QZ, TZ, LZ, and JS: data acquisition. QH and G-JJ: methodology and formal analysis. QH: writing – original draft. G-JJ and KW: writing – review and editing and funding acquisition. All authors had full access to all the data in the study and take responsibility for the integrity of the data and the accuracy of the data analysis.

## Footnotes

1
https://www.fil.ion.ucl.ac.uk/spm/

2
https://github.com/jigongjun/Neuroimaging-and-Neuromodulation

3
http://www.china-language.edu.cn/
