# Supplementary material for: Efficacy of twice-daily high-frequency repetitive transcranial magnetic stimulation on associative memory
Source: Front Hum Neurosci. 2022 Oct 12;16:973298. doi: 10.3389/fnhum.2022.973298 (PMC9596967; doi:10.3389/fnhum.2022.973298)
Supplement: Supplementary file 1 [file Data_Sheet_1.PDF]

Supplementary Results for

Efficacy of Twice-daily High-Frequency Repetitive Transcranial Magnetic Stimulation on Associative Memory

Stroop Test Performance in the Primary Study Cohort

The error rates on the Stroop task were not compared statistically among groups because the majority of participants committed few errors (Lee and Chan, 2000). In the response time dataset, one outlier was identified at baseline from the *Twice-daily(R)* group for the high-interference condition, which was removed from subsequent analysis. For the low-interference condition, ANOVA revealed no main effect of Group [*Twice-daily(R)* vs. *Twice-daily(S)*] ($F_{1,37} = 2.72$, $p = 0.11$) or Group × Time (Baseline vs. Post-treatment) interaction ($F_{1,37} = 0.36$, $p = 0.55$), but there was a main effect of Time ($F_{1,37} = 7.43$, $p = 0.009$). For the high-interference condition, again there was no significant Group × Time interaction ($F_{1,36} = 1.38$, $p = 0.25$) or main effect of Group ($F_{1,36} = 0.27$, $p = 0.61$), but there was a significant main effect of Time ($F_{1,36} = 21.43$, $p < 0.0001$) (**Fig. S1A**).

Stroop Test Performance in the Once-daily and Twice-daily Cohorts

Two outlier response times were removed from subsequent analysis, the one mentioned from the primary analysis and another from the *Once-daily(R)* group for the baseline low-interference condition. For the low-interference condition, ANOVA showed no significant Group [*Twice-daily(R)* vs. *Once-daily(R)*] × Time (baseline vs. post-treatment) interaction ($F_{1,33} = 0.38$, $p = 0.54$) or main effect of Group ($F_{1,33} = 0.16$, $p = 0.69$), but there was a significant main effect of Time ($F_{1,33} = 4.23$, $p = 0.048$). For the high-interference condition as well, there was no significant Group × Time interaction ($F_{1,33} = 0.998$, $p = 0.33$) or main effect of Group ($F_{1,33} = 0.09$, $p = 0.76$), but there was a main effect of Time ($F_{1,33} = 11.96$, $p = 0.002$) (**Fig. S1B**).

Stroop Test Performance in the Combined Cohort

Two outlier response times were removed from subsequent analysis (described above). For the low-interference condition, ANOVA showed no significant Time (baseline vs. post-rTMS) × Group [*Combined(R)* vs. *Twice-daily(S)*] interaction ($F_{1,52} = 0.03$, $p = 0.87$) or Main effect of Group ($F_{1,53} = 1.74$, $p = 0.19$), but there was a significant main effect of Time ($F_{1,52} = 5.76$, $p = 0.02$). For the high-interference condition, there was no significant Group × Time interaction ($F_{1,52} = 0.22$, $p = 0.64$) or

main effect of Group ($F_{1,53} = 0.27$, $p = 0.96$), but there was a significant main effect of Time ($F_{1,52} = 14.99$, $p = 0.0003$) (**Fig. S1C**).

Table S1 Time and error scores of Stroop tests

	Twice-daily(R) **(n = 20)**		***Twice-daily(S)*** **(n = 19)**		***Once-daily(R)*** **(N=16)**		***Combined(R) group*** **(N=36)**	
	Baseline	Post-rTMS	Baseline	Post-rTMS	Baseline	Post-rTMS	Baseline	Post-rTMS
Part A Time (s)	12.73 (0.40)	11.64 (0.40)	13.38 (0.57)	11.85 (0.55)	12.76 (0.76)	11.74 (0.59)	12.74 (0.36)	11.68 (0.34)
Error	0.05 (0.05)	0.20 (0.12)	0.32 (0.19)	0.11 (0.11)	0 (0)	0. (0)	0.03 (0.03)	0.11 (0.07)
Part B Time (s)	14.23 (0.52)	12.21 (0.57)	14.13 (0.47)	12.01 (0.54)	14.13 (0.82)	12.56 (0.66)	14.19 (0.46)	12.36 (0.42)
Error	0.25 (0.12)	0.15 (0.08)	0.32 (0.15)	0.05 (0.05)	0.13 (0.13)	0.19 (0.19)	0.19 (0.09)	0.17 (0.09)
Part C Time (s)	20.22 (1.02)	16.82 (1.15)	20.88 (1.01)	17.57 (0.88)	21.95 (1.67)	17.47 (1.44)	20.60 (0.93)	17.11 (0.89)
Error	1.10 (0.28)	0.70 (0.27)	1.1 (0.41)	0.95 (0.27)	0.50 (0.39)	0.31 (0.25)	0.83 (0.23)	0.53 (0.19)
Low interference[a]	1.50 (0.28)	0.57 (0.41)	0.75 (0.28)	0.16 (0.67)	1.06 (0.59)	0.82 (0.67)	1.31 (0.30)	0.68 (0.37)
High interference[b]	7.62 (0.90)	5.19 (0.97)	7.50 (0.66)	5.72 (0.57)	7.38 (1.24)	5.74 (1.47)	7.51 (0.74)	5.43 (0.83)

Note: Data are represented as mean (SEM). [a] Low interference refers to “Part B - Part A”; [b] High interference refers to “Part C - Part A”.

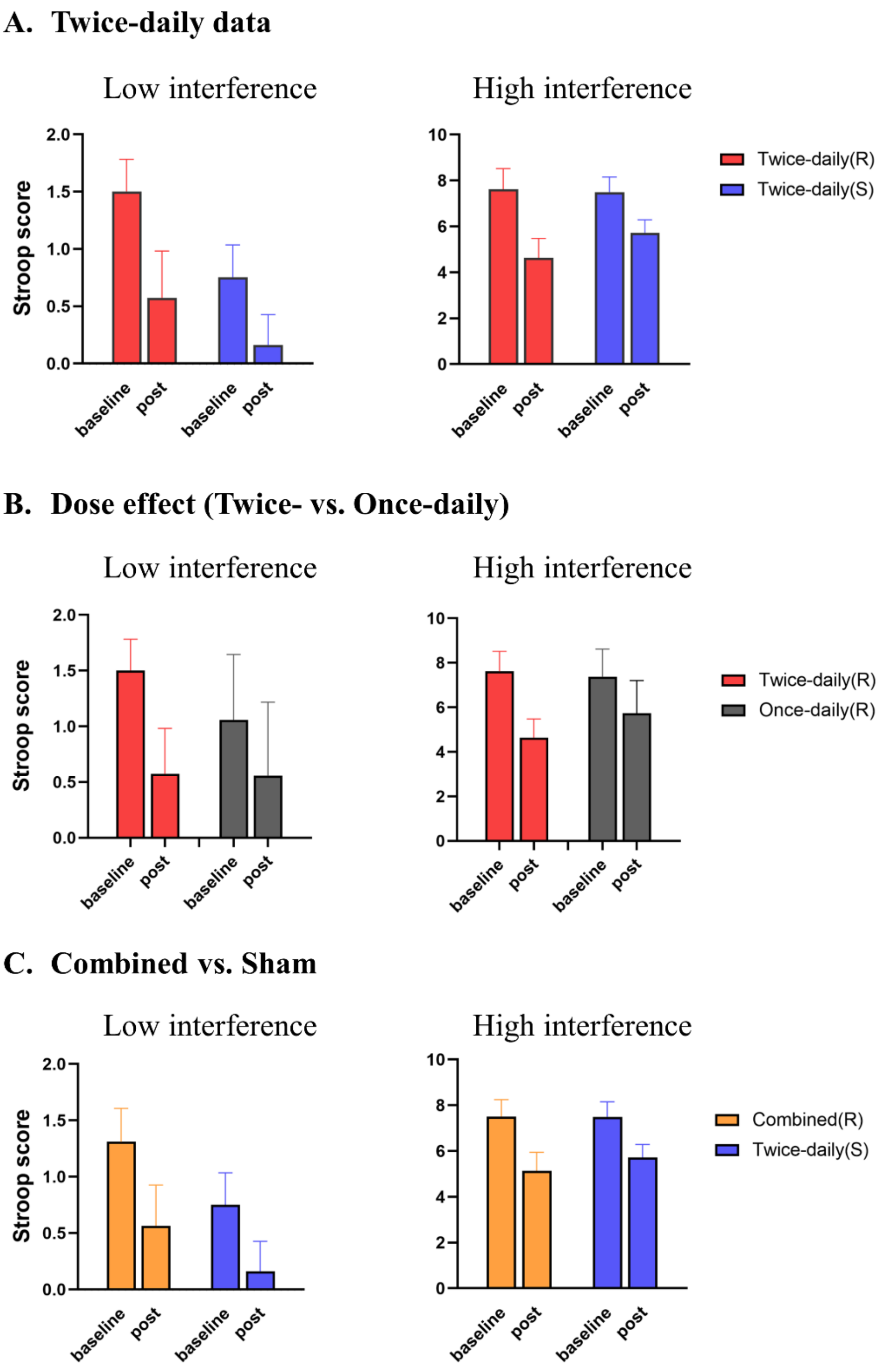

Figure S1. Effects of rTMS on Stroop task performance. There were no significant differences in Stroop task performance between (A) real and sham groups in the primary study cohort, (B) twice-daily and once-daily groups from the current and previous study cohorts, respectively, and (C) between the combined real rTMS group and twice-daily sham group.

References

Lee, T.M., Chan, C.C., 2000. Stroop interference in Chinese and English. J Clin Exp Neuropsychol 22, 465-471.
